# Comparative Calibration of Heat Flux Sensors in Two Blackbody Facilities

**DOI:** 10.6028/jres.104.030

**Published:** 1999-10-01

**Authors:** A. V. Murthy, B. K. Tsai, R. D. Saunders

**Affiliations:** Aero-Tech, Inc., Hampton, VA 23666; National Institute of Standards and Technology, Gaithersburg, MD 20899-0001

**Keywords:** heat flux, irradiance, sensor, transfer calibration

## Abstract

This paper presents the results of heat flux sensor calibrations in two blackbody facilities: the 25 mm variable temperature blackbody (VTBB) primary facility and a recently developed 51 mm aperture spherical blackbody (SPBB) facility. Three Schmidt-Boelter gages and a Gardon gage were calibrated with reference to an electrical substitution radiometer in the VTBB. One of the Schmidt-Boelter gages thus calibrated was used as a reference standard to calibrate other gages in the SPBB. Comparison of the Schmidt-Boelter gages calibrations in the SPBB and the VTBB agreed within the measurement uncertainties. For the Gardon gage, the measured responsivity in the SPBB showed a gradual decrease with increasing distance from the aperture. When the gage was located close to the aperture, a distance less than the aperture radius, the responsivity in the SPBB agreed with VTBB measurements. At a distance of about three times the aperture radius, the responsivity showed a decrease of about 4 %. This is probably due to higher convection loss from the Gardon gage surface compared to the Schmidt-Boelter sensor.

## 1. Introduction

Large aperture variable temperature blackbodies form ideal radiant energy sources for calibrating high heat flux sensors. A range of heat flux levels can be obtained at the sensor surface by varying either the source temperature or the sensor location with respect to the blackbody radiating aperture. Presently, the Optical Technology Division (OTD) at the National Institute of Standards and Technology (NIST) is developing techniques to calibrate heat flux sensors utilizing blackbody radiation. The objective is to develop the capability to calibrate sensors at flux levels up to 100 kW/m^2^. References [1] and [2] describe the blackbody radiation calibration methods in use and under development at the NIST. One of the blackbodies that has been extensively used for the calibration of heat flux sensors is the 25 mm variable temperature blackbody (VTBB). This blackbody facility is a primary facility used for radiance temperature calibrations. In this facility, heat flux sensors are routinely calibrated up to 50 kW/m^2^ flux levels with reference to an Electrical Substitution Radiometer (ESR). This technique, referred to as *transfer calibration*, has shown a long term repeatability of calibration better than 1 % in measured responsivity [3].

Further development of the capability in the VTBB to calibrate sensors at heat flux levels up to 100 kW/m^2^ would require positioning the test sensors and the reference ESR much closer to the radiating aperture. Due to the larger diameter of the ESR body compared to the radiating cavity, this would require major modifications to the facility. Further, any such modification would affect the balance of argon gas flow used for purging the high temperature graphite cavity, leading to reduced operating life of the blackbody. Hence, additional blackbody facilities are under development to extend as well as complement the present capabilities [2].

Recently, a 23 cm diameter spherical blackbody (SPBB) facility with a 50.8 mm diameter cooled aperture was commissioned. Reference [4] describes this facility and presents preliminary results of the issues involved in using this facility for both absolute and transfer calibrations. The test results in the SPBB showed the need for reducing the convection effects to make meaningful absolute calibration of sensors using blackbody radiation calculations. However, despite the strong convection effects, the results indicated that transfer calibration data of a Gardon gage with reference to a Schmidt-Boelter sensor were consistent due to similar effects of convection on both the test sensor and the reference sensor.

Following encouraging results from the preliminary transfer calibration tests in the SPBB, three other Schmidt-Boelter sensors and a Gardon gage were calibrated in both the VTBB and the SPBB to provide intercomparison data between the two facilities. Because of different radiant aperture sizes and temperature range of the two blackbodies, the intercomparison data covered different configuration factors and spectral irradiance distributions at the sensor surface. This paper gives a brief description of the experiments in the two blackbody facilities and the results of intercomparison of the sensor calibrations.

## 2. Experiments

**Fig. 1:**
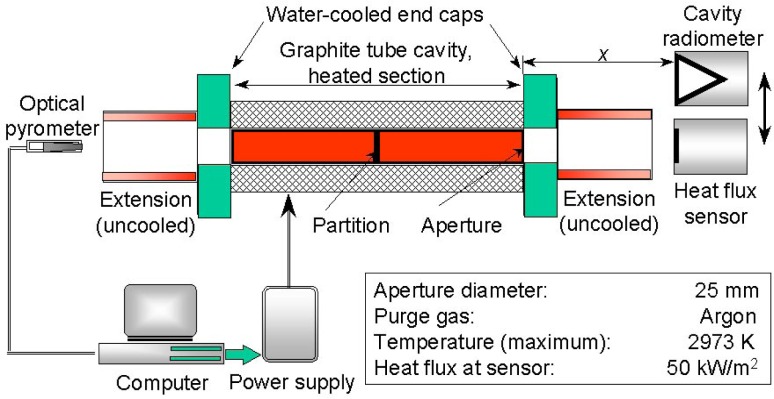
Schematic diagram of the 25 mm Variable Temperature Blackbody (VTBB). *x* represents the distance of sensor location from the blackbody aperture.

**Fig. 2:**
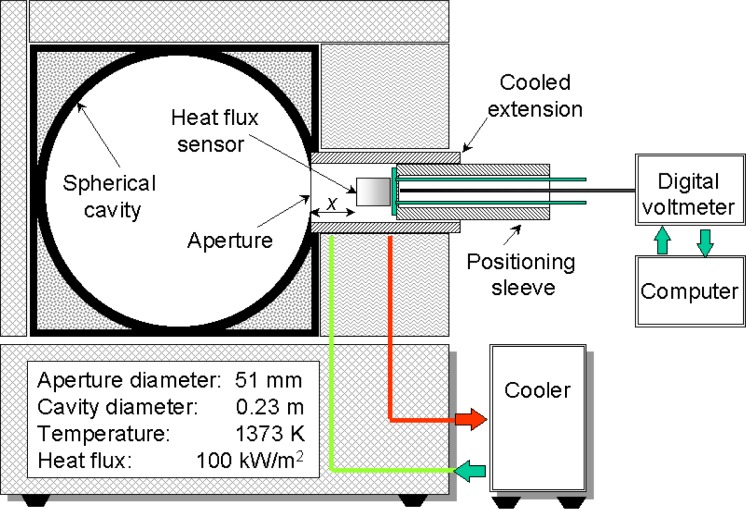
Schematic diagram of the Spherical Blackbody (SPBB) with cooled aperture. *x* represents the distance of sensor location from the blackbody aperture.

Figures 1 and 2 show the layout of the experimental setup in two blackbody facilities: the 25 mm VTBB and the SPBB, respectively. The VTBB (Thermogage Inc., Frostburg, MD)^1^ is an electrically heated 25 mm diameter graphite tube cavity blackbody furnace. The heated section is 28.2 cm long with a 3 mm center thick partition. Direct resistance heating of the graphite tube using large ac currents and low voltages provides for quick heating and cool down of the furnace. The tube end caps are water-cooled and are directly connected to the heating electrodes. The design provides a uniform temperature distribution along the cavity with a sharp temperature gradient between the water-cooled copper end cap and the graphite heater element. The apparent emissivity of the cavity is higher than 0.99. A proportional-integral-differential (PID) controller regulates the power supply to maintain the furnace temperature, measured by an optical pyrometer, to within ±0.1 K of the set value. The maximum recommended operating temperature for the furnace is 2923 K. The heat flux sensors to be calibrated and the reference radiometer are located at a fixed distance from the exit of the blackbody.

The SPBB facility was designed and developed recently to facilitate testing heat flux sensors in a cooled enclosure. The design was based on the concept first proposed by Olsson [5, 6]. Its radiating cavity is a 0.23 m diameter spherical furnace fitted with a 50.8 mm diameter aperture (fabricated by Mikron Instrument Company, Oakland, NJ). The furnace wall, made of clay and coated on the inner surface with a high temperature black paint, is electrically heated. The exterior of the furnace is air cooled. The furnace can be operated continuously up to a maximum temperature of 1373 K, and up to 1446 K for shorter duration. A PID controller maintains the cavity temperature, measured by a precision Type-S thermocouple, within ±1 K of the set value. The sensor to be calibrated is located inside a water-cooled enclosure attached to the furnace. The water-cooled enclosure is comprised of a cylindrical tube with a precision aperture at one end fitted to the radiating cavity of the furnace. The other end serves as an opening for inserting the sensor housing assembly. The inside of the tube is coated with a high temperature black paint. The cooled enclosure minimizes reflected radiation from the inner surface of the enclosure on to the sensor surface. The test sensor is located inside the enclosure at a known distance from the aperture with reference to a stop ring.

Table 1 lists the type and description of the four heat flux sensors tested in the present experiments. The Schmidt-Boelter sensor, designated SB-0, is being used as a reference gage to check the long term repeatability of calibration in the VTBB. This sensor has been calibrated several times in the VTBB.

Table 2 gives the calibration test conditions in the VTBB and the SPBB. For all the calibrations in the VTBB, a previously characterized electrical substitution radiometer (ESR) was used as a reference standard. For tests in the SBPP, one of the water-cooled Schmidt-Boelter gages (SB-1) was used as a reference for transfer calibration of the Schmidt-Boelter sensor and the Gardon gage, designated as SB-2 and GG-1, respectively. This was necessary since the ESR could not be used as a reference directly in the SPBB setup because of the mechanical constraints of placing the ESR inside the cooled enclosure close to the aperture. By using gage SB-1 as the reference, it was possible to compare the calibration in the SPBB with the direct calibration against the ESR in the VTBB.

## 3. Results and Discussion

**Fig. 3:**
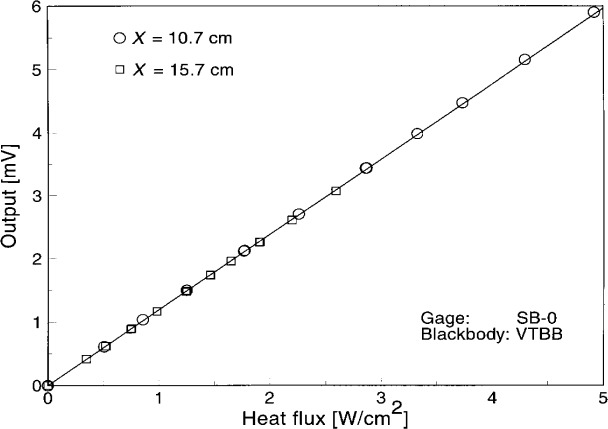
Reference Schmidt-Boelter gage calibration (SB-0) in the 25 mm Variable Temperature Blackbody (VTBB) against the electrical substitution radiometer. *x* represents the distance of sensor location from the blackbody aperture.

**Fig. 4:**
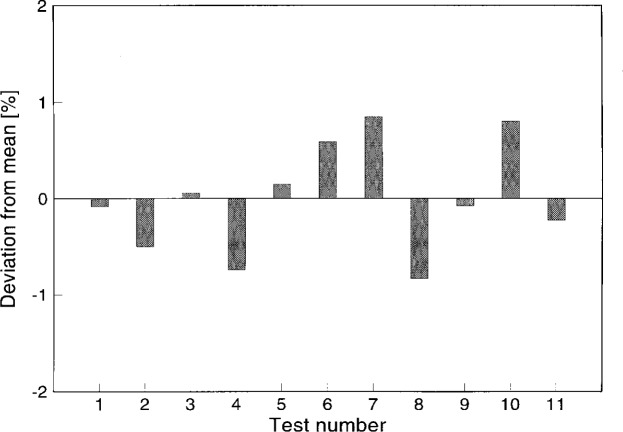
Long term repeatability of reference Schmidt-Boelter gage (SB-0) responsivity in the 25 mm Variable Temperature Blackbody (VTBB) against the electrical substitution radiometer.

First the reference Schmidt-Boelter gage SB-0 was calibrated to check the long- term repeatability of calibration in the VTBB. Figure 3 shows the results of the present repeat calibration in the VTBB using the reference transfer standard ESR. The responsivities calculated from two calibrations at distances of *x* = 10.7 cm and *x* = 15.7 cm from the VTBB aperture agree within 1 %. Figure 4 shows the long term repeatability of the calibration of this gage, covering 11 tests in the last 2 years. The variation in measured responsivity over this period is less than 1 % of the mean sensitivity of 0.119 mV/(kW/m^2^) and the corresponding relative standard deviation of the mean is about 0.2 %. This variation is within the assigned 2 % relative expanded uncertainty (coverage factor *k* = 2) of the calibration.

Following the check-out calibration on the Schmidt-Boelter gage SB-0, the other two Schmidt-Boelter gages, SB-1 and SB-2, and the Gardon gage (GG-1) were calibrated in the VTBB with reference to the transfer standard ESR. Table 3 summarizes the results of this calibration. The test data exhibited good linearity over the heat flux calibration range with regression factors of 1.000. The measured responsivities at two locations, *x* = 10.7 cm and *x* = 15.7 cm from the aperture, agreed within 0.5 % of the mean values of 0.117 mV/(kW/m^2^) and 0.070 mV/(kW/m^2^) for the SB-1 and SB-2 sensors, respectively. A similar calibration of the Gardon gage (GG-1) at *x* = 10.7 cm from the aperture in the VTBB from a previous test indicated a responsivity of 0.096 mV/(kW/m^2^).

For tests in the SPBB, the Schmidt-Boelter gage SB-1 calibrated in the VTBB was used as a reference standard. The other Schmidt-Boelter gage SB-2 and the Gardon gage GG-1 were calibrated against the reference gage SB-1. The calibration in the SPBB was done by locating the gage inside the cooled enclosure at distances of *x* = 1.27 cm, *x* = 2.91 cm, and *x* = 4.70 cm from the radiating aperture. Table 4a summarizes the results of SB-2 calibration in the SPBB. The response of the gage was linear at all three locations over the calibration heat flux range of 0 to 10 kW/m^2^. Three calibrations were done at each location. The responsivities at all the locations agreed within 1 %.

**Fig. 5:**
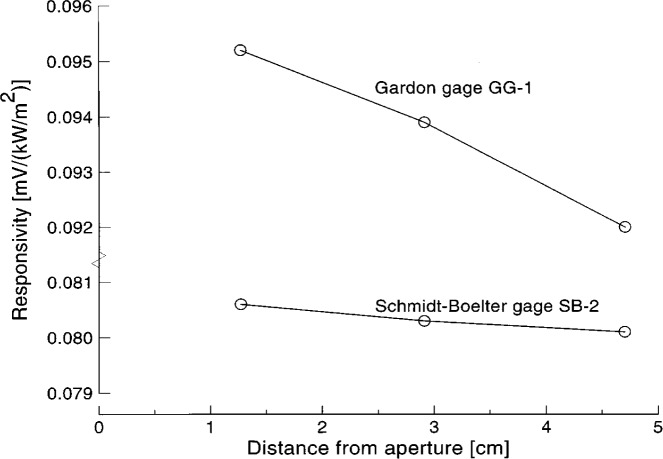
Variation in measured mean responsivity with distance from aperture in the spherical blackbody (SPBB).

Table 4b summarizes the corresponding calibrationsults for the Gardon gage GG-1. In one of the tests, the uncooled Schmidt-Boelter gage SB-0, instead of SB-1, was used as the reference standard. For this Gardon gage, the measured responsivities at each location from three tests agree within 1 %. However, the mean responsivity decreases gradually when the gage is moved away from the aperture, as shown in Fig. 5.

The reason for the decrease in responsivity with increasing distance measured in the SPBB is probably due to different principles of operation of the Gardon and Schmidt-Boelter gages. In the Gardon gage, the incident heat flux on the sensitive surface is dissipated radially to the heat sink from the center of the circular foil to the edge. The output of the gage is proportional to the temperature difference between the foil center and the heat sink. The temperature distribution across the circular foil is parabolic with a peak value of over 100 °C at higher heat flux levels. For a Schmidt-Boelter sensor, the corresponding temperature distribution is nearly uniform across the sensor surface. Because of higher peak temperatures on the Gardon gage circular foil, the heat loss can be relatively larger compared to a Schmidt-Boelter gage. As demonstrated in Ref. [4], convection effects can have a significant influence on the gage output in a cooled enclosure due to strong induced flow of the hot gas from the blackbody furnace. This effect becomes proportionately larger compared to the radiant heat flux when the sensors are at a distance of *x* = 4.70 cm from the aperture. Such convection effects are present even for Schmidt-Boelter sensors but are similar for the reference as well as the test gages in the present calibration. Further, the physical dimensions of all the Schmidt-Boelter sensors in this calibration were the same, contributing to nearly identical enclosure geometry for both the radiation and convection heat transfer at the sensor surface. In contrast, the Gardon gage has a water-cooled body diameter of 25.4 mm and hence the convection effect around the gage is likely to be different from the reference Schmidt-Boelter gage which has a body diameter of about 6 mm.

**Fig. 6:**
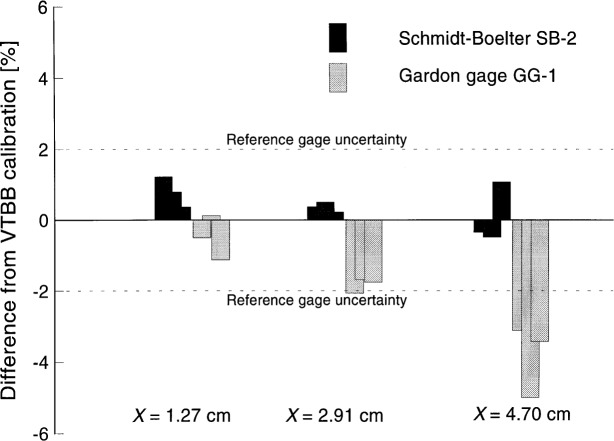
Comparison of the Schmidt-Boelter gage (SB-2) and the Gardon gage GG-1 measured responsivities in the spherical blackbody (SPBB) with reference to the 25 mm Variable Temperature Blackbody (VTBB) calibrations. *x* represents the distance of sensor location from the blackbody aperture. Dotted lines represent the relative expanded uncertainty in the reference gage (SB-0) calibration.

Figure 6 shows the comparison of the responsivity measured in the SPBB, with reference to the VTBB measurements using ESR as the transfer standard. The Schmidt-Boelter calibrations in both facilities agree within the 2 % relative expanded uncertainty (coverage factor *k* = 2) of the test. As discussed, the responsivity of the Gardon gage is within this limit for distances *x* = 1.27 cm and *x* = 2.91 cm, but drops by about 4 % at *x* = 4.70 cm.

The results of the comparative calibration suggest that the SPBB can be used for transfer calibrations in the best manner when the physical size of the gages and the enclosure geometry for the test and reference sensors are the same, and the sensors are of the same type. More tests on a different Gardon gage reference standard will be helpful to examine whether the differences observed between the two types of sensors are entirely sensor-related or facility-related. Further, it must be noted that the present tests were conducted in a completely enclosed mode, with the sensor housing fitting tightly inside the cooled extension, to obtain maximum total heat flux at the sensor surface. Significant convection effects can be present in this closed configuration due to induced flow. It would be interesting to see whether testing with a clearance around the gage holder to reduce the effects of induced flow will give better agreement for the Gardon gage at all locations. The results of Ref. [4] suggest that such a clearance around the holder is helpful in reducing the convection heating of the sensor by furnace gas.

## 4. Conclusions

Comparative transfer calibrations of Gardon and Schmidt-Boelter type heat flux sensors were conducted in two blackbody facilities: the 25 mm VTBB and a recently developed 51 mm aperture SPBB. The VTBB calibrations used an ESR as a reference transfer standard. For SPBB calibrations, a water-cooled Schmidt-Boelter gage whose calibration was derived from a transfer calibration in the VTBB with reference to an ESR was used. The results of calibrations using the two different blackbodies agreed within the test uncertainty when the test and the reference gages were both of the same Schmidt-Boelter type. For the Gardon gage, the SPBB tests using a water-cooled enclosure showed a decrease in responsivity with increasing distance from the aperture. At a distance of about three times the aperture radius, the decrease in was about 4 %. This decrease is expected to be due to higher convection loss from the Gardon gage surface compared to the Schmidt-Boelter sensor. The tests demonstrate that to perform reliable transfer calibrations in the cooled enclosure of the SPBB, it is necessary that both the gage to be calibrated and the reference standard are of same type. Also, the internal configuration of the gage mounting inside the cooled enclosure should be identical in order to keep convection effects the same on both the reference and test gage.

## Figures and Tables

**Table 1:** Specification of heat flux sensors used in calibration

Heat flux sensor type (designation)	Range (kW/m^2^)	Water cooling	Dimensions diameter	(mm) length
Schmidt-Boelter (SB-0)^a^	110	no	4.75	8.9
Schmidt-Boelter (SB-1)	110	yes	6.35	15.9
Schmidt-Boelter (SB-2)	220	yes	6.35	15.9
Gardon (GG-1)	110	yes	25.4	25.4

a Reference test gage in VTBB

**Table 2:** Calibration test conditions

Test parameter	VTBB	SPBB
Radiating aperture diameter *x* (cm)	2.54	5.08
Aperture to sensor distance *x* (cm)	10.7^a^, 15.7^a^	1.27^b^, 2.91^b^, 4.70^b^
Temperature range	1323 K to 2653 K	673 K to 1373 K
Purge gas	Argon	None
Radiating aperture emissivity	> 0.99	0.997
Heat flux range (kW/m^2^)	50	120

a Open mode (sensor located in ambient air). Distances correspond to 12.5 mm and 62.5 mm from the exit.

b Closed mode (sensor inside cooled enclosure).

**Table 3:** Calibration results from VTBB tests

Heat flux sensor type (designation)	Responsivity mV/(kW/m^2^)
Schmidt-Boelter (SB-0)	0.119
Schmidt-Boelter (SB-1)	0.117
Schmidt-Boelter (SB-2)	0.070
Gardon (GG-1)	0.096

**Table 4a:** Comparison of heat flux sensor calibration in SPBB with VTBB: Calibration of Schmidt-Boelter sensor SB-2

Test date	Distance *x* (cm)	Schmidt-Boelter reference gage	Responsivity mV/(kW/m^2^)	Difference from VTBB (%)
07/31/98	1.27	SB-1	0.0709	1.22
08/01/98	1.27	SB-1	0.0706	0.80
08/22/98	1.27	SB-1	0.0703	0.37
07/31/98	2.91	SB-1	0.0703	0.37
08/01/98	2.91	SB-1	0.0704	0.51
08/22/98	2.91	SB-1	0.0702	0.22
07/31/98	4.70	SB-1	0.0698	−0.35
08/01/98	4.70	SB-1	0.0697	−0.49
08/22/98	4.70	SB-1	0.0708	1.08

**Table 4b:** Comparison of heat flux sensor calibration in SPBB with VTBB: Calibration of Gardon gage GG-1

Test date	Distance *x* (cm)	Schmidt-Boelter reference gage	Responsivity mV/(kW/m^2^)	Difference from VTBB (%)
02/15/98	1.27	SB-0	0.0952	−0.50
08/01/98	1.27	SB-1	0.0958	0.13
08/22/98	1.27	SB-1	0.0946	−1.13
02/14/98	2.91	SB-0	0.0937	−2.07
08/01/98	2.91	SB-1	0.0941	−1.68
08/22/98	2.91	SB-1	0.0940	−1.75
03/11/98	4.70	SB-0	0.0927	−3.11
08/01/98	4.70	SB-1	0.0909	−5.00
08/22/98	4.70	SB-1	0.0924	−3.43

## Footnotes

1 Certain commercial equipment, instruments, or materials are identified in this paper to foster understanding. Such identification does not imply recommendation or endorsement by the National Institute of Standards and Technology, nor does it imply that the materials or equipment are necessarily the best available for the purpose.
